# *Staphylococcus aureus* Manganese Transport Protein C (MntC) Is an Extracellular Matrix- and Plasminogen-Binding Protein

**DOI:** 10.1371/journal.pone.0112730

**Published:** 2014-11-19

**Authors:** Natália Salazar, Mónica Marcela Castiblanco-Valencia, Ludmila Bezerra da Silva, Íris Arantes de Castro, Denize Monaris, Hana Paula Masuda, Angela Silva Barbosa, Ana Paula Mattos Arêas

**Affiliations:** 1 Centro de Ciências Naturais e Humanas, Universidade Federal do ABCSanto André, Brazil; 2 Departamento de Imunologia, Instituto de Ciências Biomédicas, Universidade de São Paulo, São Paulo, Brazil; 3 Laboratório de Bacteriologia, Instituto Butantan, São Paulo, Brazil; University of North Dakota School of Medicine and Health Sciences, United States of America

## Abstract

Infections caused by *Staphylococcus aureus* – particularly nosocomial infections - represent a great concern. Usually, the early stage of pathogenesis consists on asymptomatic nasopharynx colonization, which could result in dissemination to other mucosal niches or invasion of sterile sites, such as blood. This pathogenic route depends on scavenging of nutrients as well as binding to and disrupting extracellular matrix (ECM). Manganese transport protein C (MntC), a conserved manganese-binding protein, takes part in this infectious scenario as an ion-scavenging factor and surprisingly as an ECM and coagulation cascade binding protein, as revealed in this work. This study showed a marked ability of MntC to bind to several ECM and coagulation cascade components, including laminin, collagen type IV, cellular and plasma fibronectin, plasminogen and fibrinogen by ELISA. The MntC binding to plasminogen appears to be related to the presence of surface-exposed lysines, since previous incubation with an analogue of lysine residue, ε-aminocaproic acid, or increasing ionic strength affected the interaction between MntC and plasminogen. MntC-bound plasminogen was converted to active plasmin in the presence of urokinase plasminogen activator (uPA). The newly released plasmin, in turn, acted in the cleavage of the α and β chains of fibrinogen. In conclusion, we describe a novel function for MntC that may help staphylococcal mucosal colonization and establishment of invasive disease, through the interaction with ECM and coagulation cascade host proteins. These data suggest that this potential virulence factor could be an adequate candidate to compose an anti-staphylococcal human vaccine formulation.

## Introduction

*Staphylococcus aureus* is the causative agent of potentially harmful diseases, like necrotizing pneumonia, sepsis and endocarditis, and it is also responsible for less severe clinical manifestations such as epithelial and mucosal-associated infections. Nowadays, the emergence of methicillin (MRSA) as well as vancomycin-resistant (VRSA) strains is of great concern. MRSA variants were first described in healthcare settings but in a short period of time reached the community, presenting a more variable virulence repertoire than the susceptible counterpart strains [1]. In spite of the advances in antibiotic development, treating these infections remains a huge challenge.

Since this bacterium was isolated from a patient [2] and its effects in the host were described [3], great efforts have been made to understand and characterize virulence factors involved in the pathogenesis. In the last few years, several genomic and proteomic studies of *S. aureus* have provided countless amounts of possible targets for vaccine design [4], [5], especially those involved in the adaptation of the bacterium to host responses. A major class of proteins, responsible for the survival of the microorganism in the host, is surface proteins. They are protagonists in acquisition of cellular nutrients and in adherence to mucosal cells or the extracellular matrix (ECM), in the earlier stages of pathogenesis. They are also implicated in the invasion of sterile sites through the disruption of ECM integrity at mucosal and epithelial tissues (reviewed in [6]).

The most important staphylococcal colonization site is the nasopharynx [7]. Bacteria that infect the nasopharyngeal niche and other mucosal sites are more prone to depend on the presence of several metal ions, such as manganese, iron and zinc to colonize the host [8], [9]. As a consequence of manganese uptake, a plethora of mechanisms are activated in order to enable bacteria to survive oxidative burst in the host nasopharyngeal site. One of the most important staphylococcal enzymes responsible for oxygen detoxification is superoxide dismutase, a manganese-bound conserved protein engaged in interrupting the chain reaction triggered by superoxide (reviewed in [10]). Manganese transport protein C (MntC), another manganese binding protein, was shown to take part in this scenario in the bacterial resistance to oxidative stress [11] by competing with host calprotectin for free manganese [12].

MntC is a surface protein that is an ABC (ATP-binding cassette) transporter system component. It is widely conserved in *S. aureus*, including MRSA and VRSA strains [13]. Crystallography studies showed that this protein binds to manganese in a reversible way, by performing small changes proximal to the binding sites, composed of His, Asp and Glu residues [14].

MntC orthologs were also found in other species of the *Staphylococcus* genus, including *S. epidermidis* [13]. This species is commonly associated with asymptomatic occurrences in the community, but hospital-related infections tend to be pathogenic, possibly as a result of interspecies horizontal gene transfer events with *S. aureus*, present in healthcare settings [15].

MntC was initially annotated as a PsaA (Pneumococcal surface antigen/adhesin A) homologue, despite the relatively low similarity between these proteins' sequences. PsaA is a well-described pneumococcal virulence factor that exhibits manganese-transporter and adhesin activities [16], [17]. Several studies described the ability of PsaA to elicit a robust and protective anti-pneumococci immune response in mice [18], [19]. As its pneumococcal homologue, MntC was shown to be immunogenic and protective against *S. aureus* in a murine and an infant rat model of infection, being expressed early during the infectious process. Passive immunity due to cross-reaction was also observed against a *S. epidermidis* strain [13]. More evidence regarding the suitability of MntC as an antigen emerged from a proteomic study, which characterized many *S. aureus* phenotypes, including a MntC mutant. They showed that MntC was the only lipoprotein, highly expressed during murine and human infection, which was important to MRSA virulence [20].

Altogether, these lines of evidence suggest that MntC is more than a single surface ion-scavenging protein. A brief analysis of MntC sequence showed a significant presence of lysine residues in its composition. It could be an indicative of a plasminogen-interacting protein, similar to *Leptospira* elongation factor Tu (EF-Tu) that binds plasminogen in a lysine dependent manner [21]. Considering that during infection important virulence factors of many pathogens may interact with multiple host proteins, including ECM and coagulation cascade molecules, we evaluated whether MntC would contribute to staphylococcal colonization and dissemination by interacting with multiple ECM molecules as well as with plasminogen. *Staphylococcus aureus* possesses many factors that help it to efficiently adhere to a variety of tissues in the human host. These molecules, the microbial surface component recognizing adhesive matrix molecules (MSCRAMMs), have been studied as possible vaccine and antibiotic targets for years.

Perhaps the most characterized staphylococcal MSCRAMMs are Clumping factors (ClfA and ClfB). While the target of ClfB seems to be a protein called loricrin [22], ClfA was the first fibronectin-binding protein isolated in *S. aureus*. Cellular (insoluble) and plasma (soluble) fibronectins are broadly distributed among connective tissues. Bacterial fibronectin-binding proteins can exhibit 8 different domains, representing a key factor in cell adhesion [23]. Although ClfA possesses type I Fibronectin binding domain (FBD1), other staphylococcal proteins that interact with this ECM component, Fibronectin-binding protein A and B (FnBPA and FnBPB), present FBD1 and FBD2 in tandem, bound to FBD5 [23]. Unlike Fibronectin-binding proteins, Staphylococcal Collagen adhesin (Cna) exhibits only one type of binding domain. Recent studies showed that collagen docking occurs by a conserved mechanism detected in collagen-binding proteins called collagen hug [23].

Besides ClfA, fibrinogen-binding protein (FbI) is a MSCRAMM involved in fibrinogen-mediated cell adhesion. Both ClfA and FbI are capable of specifically binding the human fibrinogen γ-chain, but exhibit poor affinity for the bovine molecule and no capacity to interact with its ovine analogue [24]. It reflects the molecular adaptation of *S. aureus* to infect human and less frequently bovine hosts.

Laminin-binding proteins are important bacterial adhesins, since this protein is found in all types of tissues as networks, conferring a highly cross-linked character to ECM. Eighteen isoforms of laminin were isolated, until the moment, based on different forms of arranging the subunits. Maybe as a result of this variation, no laminin-domain can be found in a specific database [23]. In *S. aureus*, α-enolase is a laminin- as well as a plasminogen-binding protein [25], [26]. The multiple functions of enolase account for the underestimated role of this protein in staphylococcal virulence, since bacterial mutants would be impracticable.

While considered a minor component of ECM, elastin is a special factor that confers elastic property to the matrix. Like laminin, no elastin-binding domain was deposited in specific databases. Elastin-binding protein (EbpS) and FnBPA are responsible for elastin-mediated adhesion performed by *S. aureus* [23].

Our data supported the hypothesis that MntC could be a staphylococcal MSCRAMM and a plasminogen-binding protein. Thus, we describe a novel function for MntC that may contribute to staphylococcal pathogenesis. One could speculate that the protective role for MntC observed in animal models [13] may be due to impairment of its adhesive properties.

## Materials and Methods

### Ethics Committee Approval

Animals were supplied with food and water *ad libitum* and experimental protocols were previously approved by the Ethical Committee for Animal Research of the Biomedical Sciences Institute, University of São Paulo, São Paulo, Brazil, under the license number 061/10/CEUA.

### Bacterial strains, culture and plasmids

*Staphylococcus aureus* strain ATCC 25923 was cultured for 16 h at 37°C, under aerobic conditions, in liquid BHI medium (Himedia). Cells were harvested by centrifugation at 5000×*g* for 10 min and resuspended in enzymatic lysis buffer 20 mM Tris.Cl, pH 8.0, 2 mM sodium EDTA, 1.2% Triton X-100. Immediately before use, lysozyme (20 mg/mL) was added. For purification of DNA the DNeasy Blood & Tissue Kit (Qiagen) was used, according to manufacturer's instructions. *Escherichia coli* DH5α was used as the cloning host strain and *E. coli* BL21 (DE3) was utilized for the expression of the recombinant protein, using the T7 promoter based pRSET-C (Invitrogen) expression plasmid.

### Purified proteins and antibodies

All macromolecules from the extracellular matrix (ECM) were purchased from Sigma-Aldrich. Laminin-1 and collagen type IV were derived from the basement membrane of Engelbreth-Holm-Swarm mouse sarcoma, cellular fibronectin was derived from human foreskin fibroblasts, and plasma fibronectin was isolated from human plasma. Fibrinogen and plasminogen were isolated from human plasma. Mouse monoclonal anti-human fibrinogen α-chain was purchased from BD Biosciences and secondary peroxidase-conjugated antibodies from Sigma-Aldrich.

### Cloning, expression, purification of recombinant proteins and generation of antiserum

The *mntC* gene was amplified by PCR from genomic DNA of *S. aureus* ATCC 25923 using the primers: F: 5′ - GAATTCGTTATTTCATGCTTCCGTGTACAGT - 3′/R: 5′ - GGATCCGAGGTACTGGTGGTAAACAAAGCAG - 3′. PCR fragments were cloned into pGEM T-Easy vector (Promega) and transformed into *E*. *coli* DH5α. Following digestion with the restriction enzymes *Eco*RI and *Bam*HI, fragments were subcloned into the *E. coli* expression vector pRSET-C. The *E. coli* BL21 (DE3) cells transformed with pRSET-C/*mntC* were grown for 16 h at 30°C in 250 mL of LB (Luria-Bertani medium) with 100 µg/ml ampicillin. Culture was grown until an optical density at 600 nm of 0.8 was observed, and IPTG (1 mM) was added. After 3 h of incubation, the cells were harvested by centrifugation, and the bacterial cell pellet was resuspended in a solution containing 20 mM sodium phosphate (pH 7.4), 100 mM NaCl, and lysed in a sonicator. Aliquots of total cellular extracts were collected and analyzed by 12% sodium dodecyl sulfate polyacrylamide gel electrophoresis (12% SDS–PAGE). The his-tagged protein was purified using the AKTA purifier 10 system (GE Healthcare). The suspension was loaded onto a Ni^2+^-charged chelating Sepharose HisTrap HP (GE Healthcare). Contaminants were washed away with a solution containing 20 mM sodium phosphate (pH 7.4), 500 mM NaCl and 20 mM imidazole. The recombinant protein was then eluted with a solution containing 20 mM sodium phosphate (pH 7.4), 500 mM NaCl and increasing amounts of imidazole from 20 to 500 mM. The protein was extensively dialyzed against phosphate-buffered saline (PBS) at 4°C for 48 h. Purified protein samples were analyzed by 12% SDS–PAGE. The His-tag was removed by cleavage with enterokinase (New England Biolabs). The solution was then loaded onto a strong cation exchanger Sepharose HiTrap FF (GE Healthcare) in the AKTA system mentioned above in order to remove enterokinase, according to the manufacturer's specifications. LIC10301, LigBC and EF-Tu *Leptospira* control proteins were expressed and purified as previously described [27], [28], [21].

### Circular dichroism spectroscopy

Purified recombinant MntC was dialyzed against sodium phosphate buffer (pH 7.4). Circular dichroism (CD) spectroscopy measurements were performed at 20°C using a Jasco J-810 spectropolarimeter (Japan Spectroscopic) equipped with a Peltier unit for temperature control. Far-UV CD spectra were measured using a 1-mm-path-length cell at 0.5-nm intervals. The spectrum was presented as an average of five scans recorded from 190 to 260 nm.

### Antisera against recombinant proteins

Ten female BALB/c mice (4 to 6 weeks old) were immunized by intraperitoneal route with 10 µg of recombinant proteins. Aluminum hydroxide was used as adjuvant. Two subsequent booster injections were given at 2-week intervals with the same protein preparation. Negative control mice were injected with PBS. The mice were bled from the retro-orbital plexus and the pooled sera were analyzed by enzyme-linked immunosorbent assay (ELISA) for determination of antibody titers.

### Binding of MntC to ECM and coagulation cascade molecules

Protein attachment to individual macromolecules of the extracellular matrix was analyzed according to a previously published protocol [29] with some modifications. Briefly, ELISA plate wells (Nunc-Immuno plate, MaxiSorp surface) were coated with 1 µg of laminin, collagen type IV, cellular fibronectin, plasma fibronectin, plasminogen and fibrinogen in 100 µL of PBS followed by a 16–20 h incubation at 4°C. The wells were washed three times with PBS–0.05% Tween 20 (PBS-T) and then blocked with 200 µL of 1% BSA for 2 h at 37°C. For determination of dose-dependent attachment, protein concentrations varying from 0 to 2 µM were added per well in 100 µL of PBS and proteins were allowed to adhere to the different substrates for 1 h 30 min at 37°C. After washing six times with PBS-T, bound proteins were detected by adding 100 µL of adequate dilutions of the respective mouse antisera in PBS. Incubation proceeded for 1 h, and after three washes with PBS-T, 100 µL of a 1∶5000 dilution of horseradish peroxidase-conjugated goat anti-mouse immunoglobulin G (IgG) (Sigma-Aldrich) in PBS were added per well for 1 h. All incubations took place at 37°C. The wells were washed three times, and *o*-phenylenediamine (0.04%) in citrate phosphate buffer (pH 5.0) plus 0.01% (wt/vol) H_2_O_2_ was added. The reaction was allowed to proceed for 15 min and was then interrupted by the addition of 50 µL of 4 M H_2_SO_4_. The absorbance at 492 nm was determined in a microplate reader (Labsystems Uniscience Multiskan EX). LigBC and LIC10301, both from *Leptospira interrogans*, were used as positive and negative controls, respectively. LigBC is a surface lipoprotein previously shown to bind multiple ECM macromolecules [30]–[37]. LIC10301 does not bind to any ECM molecule used in the present study [21]. Two independent experiments were performed, each one in duplicate. To determine the role of lysines and ionic strength in MntC plasminogen interactions, ELISA plate wells were coated with plasminogen (10 µg/mL). The same protocol mentioned above was followed except that ε-aminocaproic acid (0–10 mM) or NaCl (0–400 mM) was added with recombinant MntC (10 µg/mL) to plasminogen-coated wells. Bound MntC was detected with mouse polyclonal anti-MntC at a proper dilution followed by horseradish peroxidase-conjugated goat anti-mouse immunoglobulin G (IgG) at a 1∶5000 dilution. Student's two-tailed *t* test was used for statistical analysis. A *p* value less than 0.05 was considered statistically significant.

### Plasmin activity after plasminogen activation

Microtiter plate wells were coated with recombinant proteins (10 µg/mL) and BSA (nonglycosylated attachment-negative control protein). After blocking with 3% BSA diluted in PBS, plasminogen (20 µg/mL) was added and incubation proceeded for 1 h at 37°C. Unbound plasminogen was removed by washing wells three times with PBS-T, and then human urokinase plasminogen activator (uPA, Sigma-Aldrich) (3 U/well) and the chromogenic substrate D-valyl-leucyl-lysine-ρ-nitroanilide dihydrochloride (25 µg/well, Sigma-Aldrich) dissolved in PBS were added. The plates were incubated at 37°C and absorbance at 405 nm was read after 24 h. *Leptospira interrogans* EF-Tu was used as a positive control, previously shown to bind plasminogen [21].

### Fibrinogen degradation assay

Recombinant proteins (10 µg/mL) and BSA were immobilized onto microtiter plate wells. EF-Tu was used as positive control. After blocking with 3% BSA diluted in PBS, plasminogen (20 µg/mL) was added and incubation proceeded for 1 h at 37°C. Wells were washed with PBS-T and human fibrinogen (10 µg or 500 ng, plasminogen depleted; Calbiochem) together with plasminogen activator uPA (3 U) were added. Reaction mixtures were incubated at 37°C for the indicated time points, and were then separated by 12% SDS-PAGE and transferred to polyvinylidene difluoride membranes or nitrocellulose membranes. The degradation products of fibrinogen were detected by staining the polyvinylidene difluoride membranes with Comassie Blue or by Western blotting using a mouse monoclonal antihuman fibrinogen α-chain (1∶3000) and the corresponding secondary horseradish phosphatase-conjugated antibodies. Membranes were developed with SuperSignal West Pico (Pierce).

## Results

### Expression and purification of MntC

**Figure 1:**
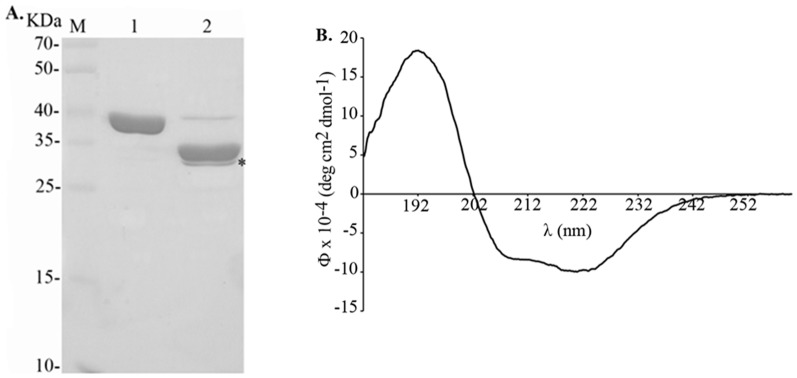
Purification and circular dichroism of recombinant MntC. (A): 12% SDS-PAGE showing purified recombinant protein MntC (1) and after removal of N-terminal polyhistidine tag (2) by enterokinase (asterisk); molecular mass marker (M). (B) MntC circular dichroism spectrum: predominant α-helical secondary structure is shown. Far-UV CD spectrum is presented as an average of five scans recorded from 182 to 262 nm; 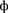, molar ellipticity.

Recombinant MntC was expressed in *E*. *coli* BL21 (DE3) in the soluble fraction. The protein was purified by Ni^2+^-charged chelating Sepharose in a single-step chromatography, and migrated as a single major band at an apparent molecular weight of 38 kDa, indicating that most of the contaminants had been removed (Fig. 1A, lane 1). The His-tag was successfully cleaved off from the purified protein after digestion with enterokinase (Fig. 1A, lane 2). The observed mobility of the poly-his tag-digested protein corresponds to its calculated molecular mass (34 kDa). Enterokinase was removed by cation exchange Sepharose chromatography (data not shown). Structural integrity of the purified protein was assessed by CD spectroscopy. As depicted in Fig. 1B, the minima at 208 and 222 nm and the maximum at 192 nm in the spectrum showed the high α-helical secondary structure content of the recombinant protein. Experimental data confirmed the secondary structure content previously predicted by three-dimensional structure of the protein solved by X-ray crystallography [14].

### MntC interacts with ECM components and coagulation cascade molecules

**Figure 2:**
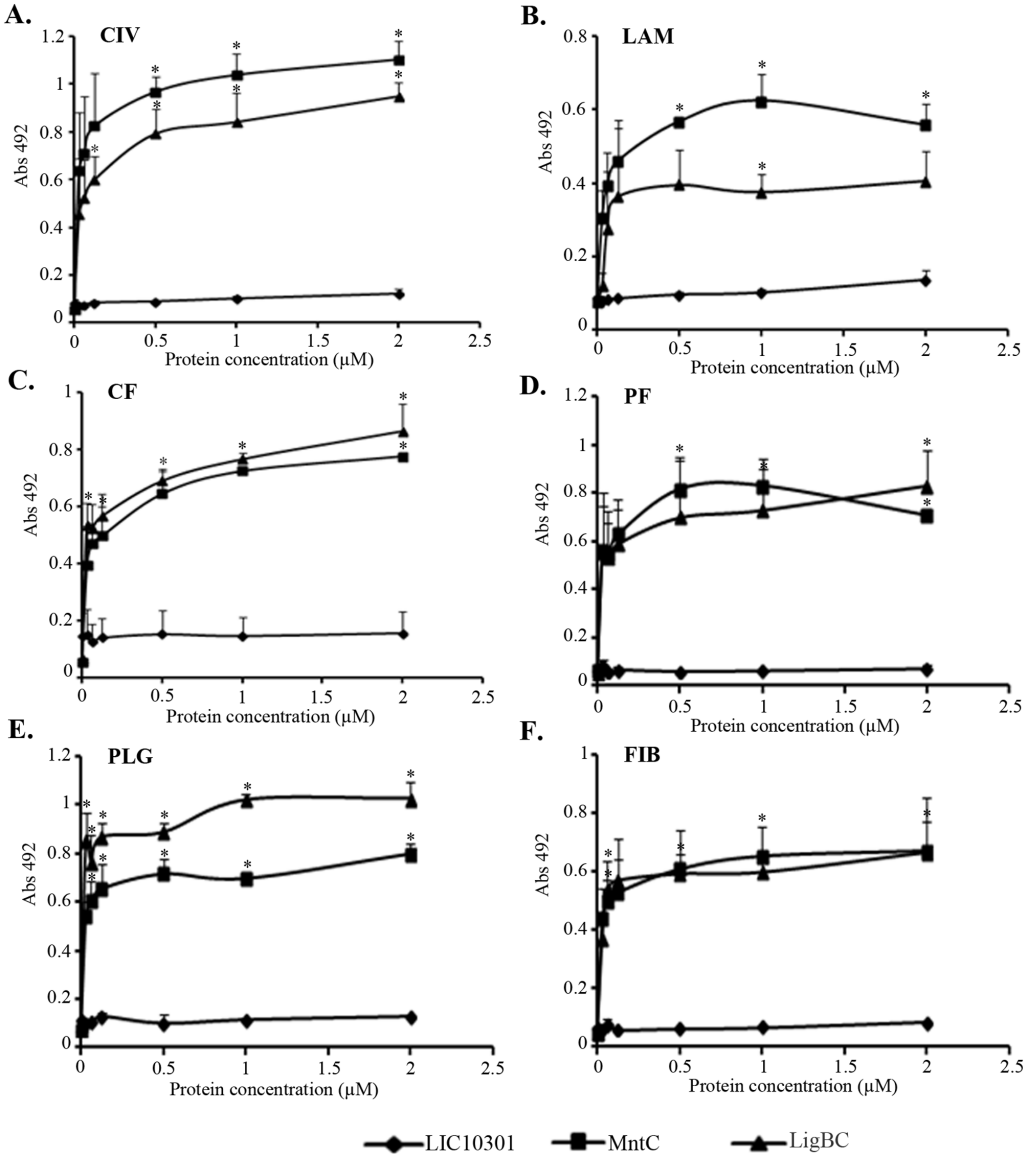
Binding of MntC to ECM components as a function of protein concentration. (A) Collagen type IV (CIV), (B) laminin (LAM), (C) cellular fibronectin (CF), (D) plasma fibronectin (PF), (E) plasminogen (PLG), (F) fibrinogen (FIB). LigBC and LIC10301 were included as positive and negative controls, respectively. Recombinant protein concentrations ranged from 0 to 2 µM. Each point represents the mean absorbance value at 492 nm ± the standard error of two independent experiments, each performed in duplicate. MntC binding to each ECM component was compared to LIC10301 binding to these molecules by the two-tailed t test (* *p*<0.05).

Since MntC is a cell surface protein capable of eliciting protective immunity against *S. aureus* and *S. epidermidis* [13], we investigated its ability to interact with host molecules such as ECM and coagulation cascade components. Collagen type IV, laminin, cellular fibronectin, plasma fibronectin, plasminogen and fibrinogen were immobilized on microtiter wells, and recombinant protein attachment was assessed. From this assay we conclude that MntC binds all macromolecules tested in a dose-dependent and saturable manner (Fig. 2). The adhesion profiles displayed by MntC were similar to those presented by LigBC, our positive control, previously shown to bind multiple ECM macromolecules [30]–[37]. The apparent *K_d_* for MntC-collagen type IV binding is 26±12 ηM, for MntC-laminin binding is 32±11 ηM, for MntC-cellular fibronectin binding is 36±14 ηM, for MntC-plasma fibronectin binding is 18±10 ηM, for MntC-plasminogen binding is 13±5 ηM and for MntC- fibrinogen binding is 18±8 ηM. For comparison, the activity of ECM– binding from the well-characterized LigBC of *Leptospira interrogans*, is also shown; its estimated *K_d_* in this assay is 42±17 ηM for collagen type IV-LigBC binding, 42±29 ηM for laminin-LigBC binding, 23±8 ηM for cellular fibronectin-LigBC binding, 17±14 ηM for plasma fibronectin-LigBC binding, 7.4±4.1 ηM for plasminogen-LigBC binding and 19±13 ηM for fibrinogen- LigBC binding. No specific binding to the target molecules was observed when we used negative control protein LIC10301 (Fig. 2).

### Role of lysine residues and ionic strength in MntC binding to plasminogen

**Figure 3:**
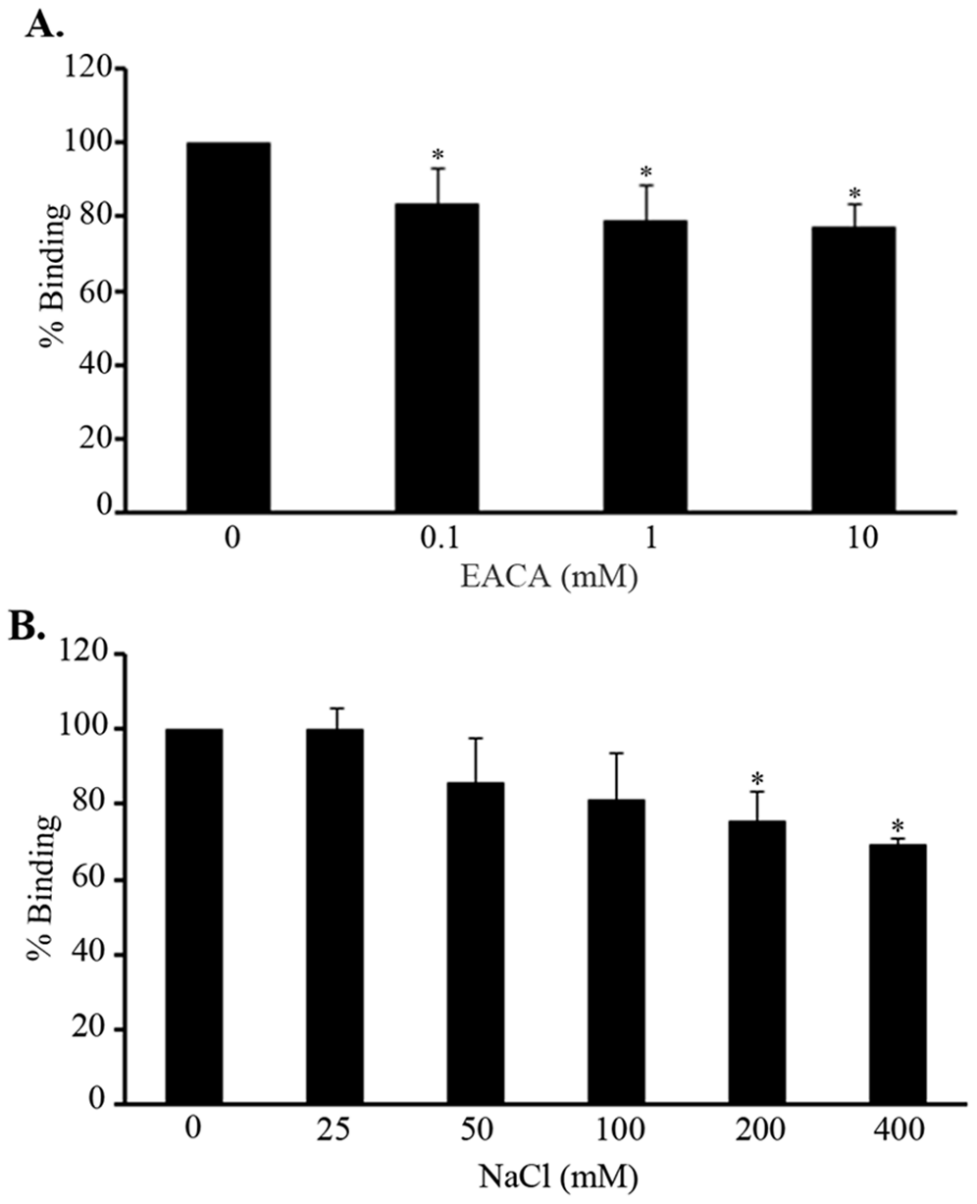
Role of lysines and salt in MntC-plasminogen interaction. MntC (10 µg/mL), in the presence (0.1–10 mM) or absence of ε-aminocaproic acid (A) and in the presence (25–400 mM) or absence of NaCl (B), was added to plasminogen-coated wells. Bound MntC was detected with a specific polyclonal antibody followed by peroxidase-conjugated anti-mouse IgG. Data represent the mean absorbance value at 492 nm ± the standard deviation of three independent experiments, each performed in duplicate. For this analysis, a Student's t-test was used (* *p*<0.05).

Several bacterial surface proteins interact with plasminogen through their lysine residues [38]. Since MntC is a lysine-rich protein, we wondered if its interaction with plasminogen would be affected by the lysine analog ε-aminocaproic acid. Apparently, lysine residues contribute to plasminogen-MntC interactions as the lysine analog partially inhibited MntC binding (Fig. 3A). However, given the low degree of inhibition observed (10–15%), other structural determinants seem to be more relevant for binding. To assess the role of ionic strength in MntC-plasminogen interactions, assays were performed in the presence of increasing amounts of sodium chloride. Salt concentrations greater than 200 mM inhibited MntC binding showing that ionic strength may also be relevant to plasminogen-MntC interactions (Fig. 3B).

### MntC-bound plasminogen is activated to plasmin

**Figure 4:**
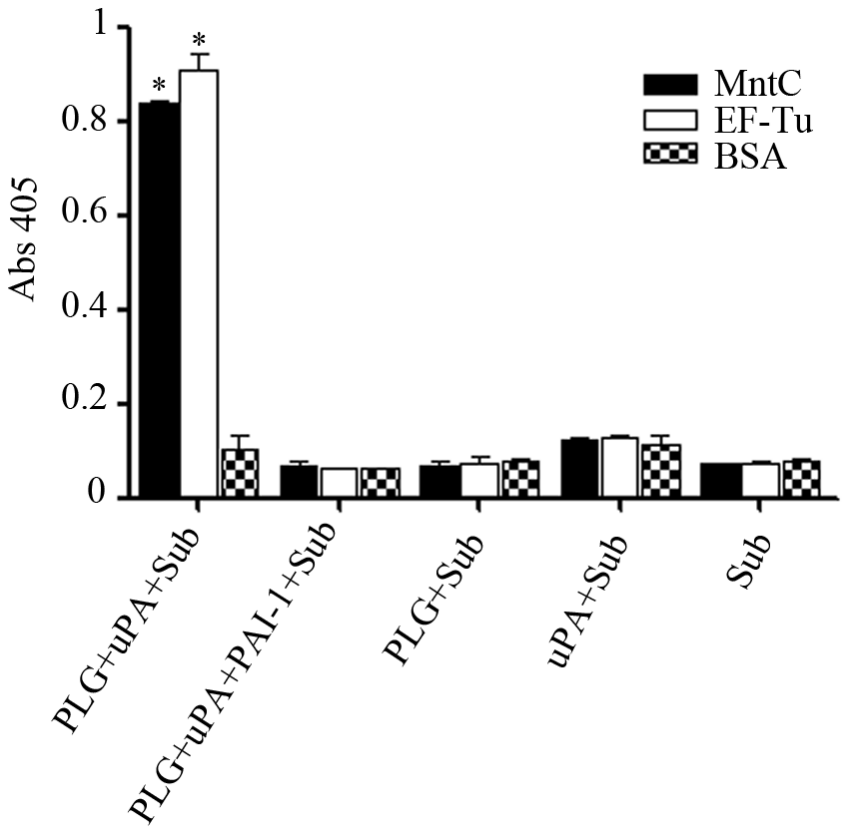
MntC-bound plasminogen is converted to functionally active plasmin. Recombinant proteins or BSA (10 µg/mL), immobilized on microtiter plate wells, were incubated with plasminogen (20 µg/mL). After washing, uPA (3 U) and the chromogenic substrate D-valyl-leucyl-lysine-ρ-nitroanilide dihydrochloride (25 µg/well) were added. Data represent the mean absorbance value at 405 nm ± the standard deviation of two independent experiments, each performed in duplicate. For this analysis, a Student's t-test was used (* *p*<0.05). PAI-1 (plasminogen activator inhibitor 1).

To assess if MntC-bound plasminogen could be converted to active plasmin by exogenously supplied uPA, immobilized MntC was incubated with plasminogen. After extensive washing, uPA and the chromogenic substrate D-valyl-leucyl-lysine-ρ-nitroanilide dihydrochloride were added. The newly generated plasmin was able to cleave the chromogenic substrate (Fig. 4). *Leptospira interrogans* EF-Tu, previously shown to bind plasminogen [21], was used as a positive control. No cleavage of the substrate was observed in the presence of plasminogen activator inhibitor 1 (PAI-1) or in the absence of uPA, plasminogen or both uPA and plasminogen (Fig. 4).

### Plasmin bound to MntC cleaves fibrinogen

**Figure 5:**
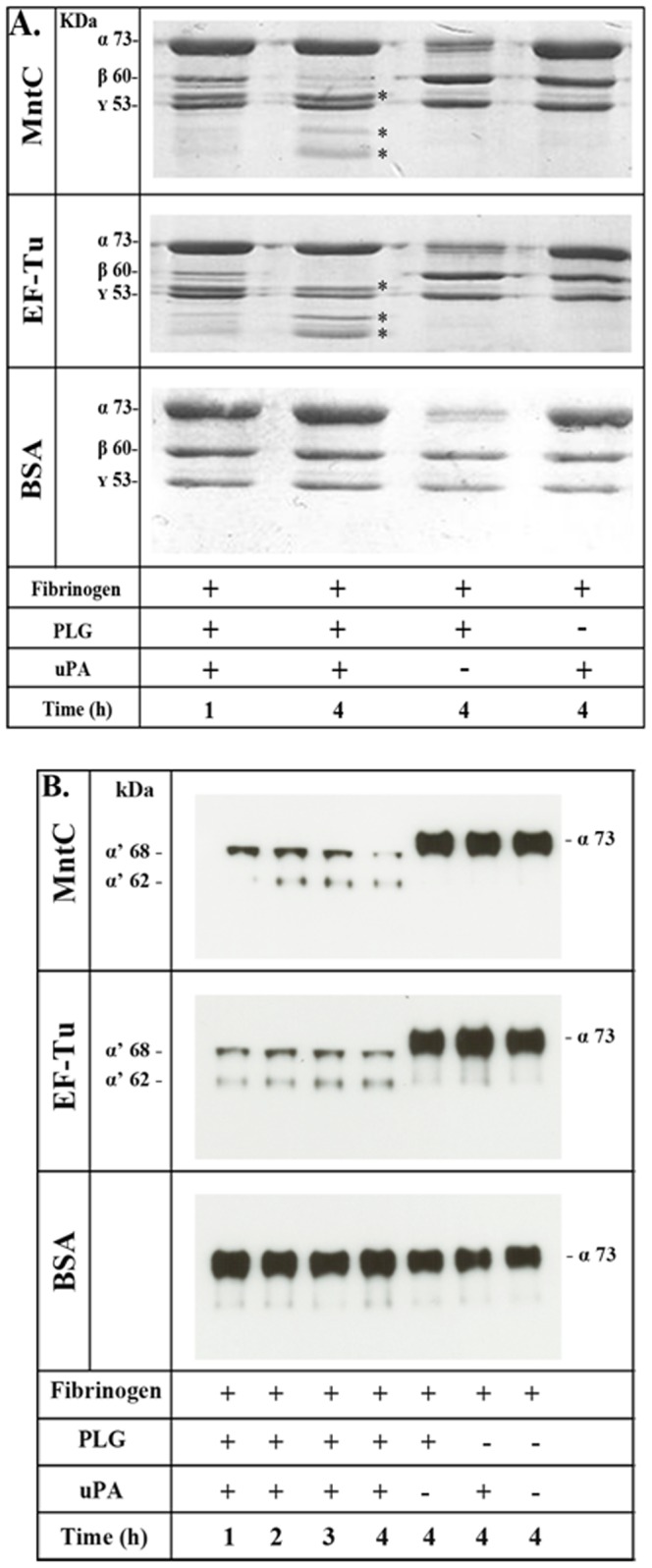
Degradation of human fibrinogen by plasmin(ogen) bound to immobilized MntC. Plasminogen (20 µg/mL) was added to immobilized recombinant proteins (10 µg/mL). After washing, fibrinogen (10 µg or 500 ng) and uPA (3 U) were added, and incubation proceeded for the indicated time points. Samples were separated by SDS-PAGE, transferred to a polyvinylidene difluoride membrane and further stained with Coomassie Blue (A) or transferred to a nitrocellulose membrane, and probed with a mouse monoclonal antibody recognizing the fibrinogen α-chain followed by the corresponding secondary HRP-conjugated antibodies (B). BSA was also included as negative control. Controls omitting uPA and/or plasminogen were included.

It is well known that the serine protease plasmin plays a crucial role in fibrinolysis and is capable of degrading extracellular matrix components. We then assayed whether MntC-bound plasmin(ogen) was able to cleave fibrinogen, one of its physiological substrates. According to our results, MntC-bound plasmin(ogen) was able to degrade fibrinogen as efficiently as EF-Tu-bound plasmin(ogen), included as a positive control (Fig. 5A and 5B). The fibrinogen α- and β-chains were degraded in a time-dependent manner, and cleavage was almost complete after 4 hours of incubation. In the absence of plasminogen or uPA, no fibrinogen degradation was observed.

## Discussion

Staphylococci may cause severe human disease, and there are no currently available vaccines to prevent infection by those bacteria. Even if one could say that a specific anti-staphylococcal vaccine is not needed, it is well established that pre-existing antibodies, specific to staphylococcal proteins, have poor opsonophagocytic as well as neutralizing activities [reviewed in [39]). This means that, in the overwhelming majority of infectious episodes in humans, previous contacts do not provide protection against staphylococcal diseases. *S. aureus* is presently considered a reemerging pathogen due to the spread of antibiotic resistant-strains into the community as well as the implementation of vaccines targeted to bacterial natural competitors of *S. aureus*, such as *Haemophilus influenzae* and *Streptococcus pneumoniae* [40]. In addition, administration of a live attenuated influenza nasal vaccine in mice was shown to affect nasopharyngeal colonization dynamics, resulting in increased *S. aureus* and *S. pneumoniae* carriage in the upper respiratory tract [41]. These studies confirm that staphylococcal infections represent a growing threat in the vaccines and antibiotics era.

MntC, a highly conserved staphylococcal surface protein, has been proposed as a potential vaccine candidate since it can provide protection in preclinical models of infection [13]. As already mentioned, MntC was initially annotated as a PsaA homologue, a pneumococcal antigen that exhibits manganese-transporter and adhesin activities [16], [17]. To further characterize the biological functions of MntC, we investigated if this surface exposed protein could interact with host molecules, notably with extracellular matrix and coagulation cascade components.

Efficient colonization of target organs by pathogenic microorganisms is achieved by their capacity to escape host innate immune responses and by their ability to interact with host cells or with the extracellular matrix [42], [43]. Extracellular matrix fills all types of tissue and connects cells through specific interactions comprising ECM components and cell receptors, such as integrins. ECM composition and fluidity vary widely among tissues. In cartilage, ECM is composed basically by intricate supramolecular collagen fibrils, on the other hand, ECM is very fluid in blood or lymph tissues, consisting mostly of plasma fibronectin [reviewed in [23]). For this reason, a bacterial protein that could efficiently bind to many components of the ECM, like MntC, would be a strategic MSCRAMM engaged in promoting bacterial adhesion to very different types of tissues. This feature could be especially important to *S. aureus*, a versatile pathogen that can potentially cause diseases in very different organs throughout the human host.

Proteolytic activity is crucial to enhance bacterial dissemination within a host. A number of microorganisms of medical importance, including *Staphylococcus aureus* [44], [45], acquire plasminogen and exploit plasmin proteolytic activity to aid tissue penetration and invasion during infection. The invasive stage of pathogenesis is commonly preceded by a permanence of the microorganism in mucosal cells. The persistence in these cells is an approach of *S. aureus* to avoid host defense mechanisms and a strategy to prepare to invasion. The bacterial entrance is mediated by an initial attachment to extracellular matrix molecules [46]. After internalization, the microorganism can replicate in a reasonable level. Later on cellular death, *S. aureus* can cleave ECM components and further, cause invasive diseases with greater bacterial load.

According to our results, MntC mediates interaction with collagen type IV, laminin, cellular and plasma fibronectin, fibrinogen and plasminogen. A dose-dependent specific and saturable binding of MntC to immobilized ECM components was observed. In addition, the *Kd* values observed for the interactions involving MntC and protein partners showed a high affinity binding. All of these features fulfill the properties of typical receptor-ligand interactions. We then explored in more details the interaction between MntC and plasminogen, a 92-kDa glycoprotein that is a key component of the host fibrinolytic system.

Plasminogen is the inactive zymogen form of plasmin. Under physiological conditions, it is converted to plasmin through cleavage at Arg^561^-Val^562^ by tissue plasminogen activator (tPA) or urokinase plasminogen activator (uPA) (reviewed in [47]). Active plasmin has a broad specificity targeting a variety of substrates, including fibrin, fibrinogen, complement C3 and C5, vitronectin, osteocalcin, factors V, VIII and X, protease-activated receptor 1, injury-induced aggregated proteins and some collagenases (reviewed in [47]). From its N terminus to its C terminus, plasminogen consists of an 80-residue sequence followed by five domains homologous to the two kringle domains of prothrombin. Both plasminogen and plasmin harbor binding sites for lysine and its analogues ε-aminocaproic acid and tranexamic acid [48]. A role for lysine residues, present in bacterial surface proteins, in plasminogen binding has been reported [44], [45], [49], [21]. In the current study, addition of ε-aminocaproic acid reduced the interaction between MntC and plasminogen, thus suggesting a role for lysines in this process. Lysine residues are highly represented in MntC protein, accounting for 15% of its total amino acid content. However, we did not observe a prominent reduction of plasminogen binding to MntC in the presence of EACA, which strongly suggests that other structural determinants may also be involved in this interaction. Ionic interactions also seem to play a role in MntC-plasminogen interaction, since concentrations of 200–400 mM NaCl did affect binding, although the extension of these ionic interactions in a physiological context remains undetermined.

Once bound to MntC, plasminogen is converted to functionally active plasmin, which, in turn, is able to degrade host fibrinogen. It is well known that *S. aureus* secretes an endogenous plasminogen activator, staphylokinase (SAK), which converts plasminogen to plasmin [25], [50]. Staphylococcal immunoglobulin-binding protein (Sbi) and extracellular fibrinogen-binding molecule (Efb) have been shown to bind Complement C3/C3b and plasminogen simultaneously. By the action of SAK, Sbi/Efb-bound plasminogen is converted to plasmin which, in turn, cleaves C3 and C3b, thus contributing to staphylococcal immune evasion [51]. In the present work, MntC-bound plasminogen was converted to active plasmin by exogenously supplied uPA. However, it is plausible that in a physiological context, activation of MntC-bound plasminogen to plasmin could also be mediated by SAK. Staphyloccocal enolase and triosephosphate isomerase (TPI), two glycolytic enzymes that serve multiple functions, have also been shown to interact with host plasminogen when displayed on the bacterial surface [25], [52]. Interestingly, while the former activates plasminogen, the latter decreases the conversion of plasminogen to plasmin, thus inhibiting the process of fibrinolysis [52].

The versatility of *S. aureus* as a pathogen derives mostly from redundant mechanisms to survive and establish a pathogenic process in the host. Genomic studies also show the presence of prophages and pathogenicity islands that apparently accounts for the higher efficiency to cause diseases than other staphylococci (reviewed in [53]). This redundancy and versatility, which enable *S. aureus* to easily adapt to host response, also extends to adhesins, specially, MSCRAMMs, as reviewed in the Introduction.

It is impressive the arsenal of MSCRAMMs that *S. aureus* uses during its pathogenesis. Redundant mechanisms are frequently observed in staphylococcal infections. It is a way to avoid specific host effectors that could potentially impair its pathogenic program. In this context, MntC seems to be a versatile MSCRAMM and plasminogen-binding protein that probably contributes to staphylococcal pathogenesis in synergy with other staphylococcal MSCRAMMs and plasminogen-binding proteins.

## References

[1] Ruffing U, Akulenko R, Bischoff M, Helms V, Herrmann M, et al (2012) Matched-cohort DNA microarray diversity analysis of methicillin sensitive and methicillin resistant *Staphylococcus aureus* isolates from hospital admission patients. PLoS ONE 7: e52487. 23285062 10.1371/journal.pone.0052487 PMC3527544

[2] Ernst HC (1896) Preliminary description of the *Staphylococcus aureus liquefacians* . J Boston Soc Med Sci 1: 3–4. PMC2121684 19971094

[3] Morse JL (1896) A study of the charges produced in the kidneys by the toxins of the *Staphylococcus pyogenes aureus* . J Exp Med 1: 613–622. 10.1084/jem.1.4.613 PMC2117933 19866816

[4] Kuroda M, Ohta T, Uchiyama I, Baba T, Yuzawa H, et al (2001) Whole genome sequencing of meticillin-resistant *Staphylococcus aureus* . Lancet 357: 1225–1240. 11418146 10.1016/s0140-6736(00)04403-2

[5] Otto A, Van Dijl JM, Hecker M, Becher D (2014) The *Staphylococcus aureus* proteome. International Journal of Medical Microbiology 3042: 110–120. 10.1016/j.ijmm.2013.11.007 24439828

[6] Foster TJ, Geoghegan JA, Ganesh VK, Höök M (2014) Adhesion, invasion and evasion: the many functions of the surface proteins of *Staphylococcus aureus* . Nature Reviews Microbiology 12: 49–62. 24336184 10.1038/nrmicro3161 PMC5708296

[7] Johannessen M, Sollid JE, Hanssen AM (2012) Host and microbe determinants that may influence the success of *S. aureus* colonization. Frontiers in Cellular and Infection Microbiology 4: 2–56. 10.3389/fcimb.2012.00056 PMC3417514 22919647

[8] Gupta R, Bhatty M, Swiatlo E, Nanduri B (2013) Role of an iron-dependent transcriptional regulator in the pathogenesis and host response to infection with *Streptococcus pneumoniae* . PLoS ONE 8: e55157. 23437050 10.1371/journal.pone.0055157 PMC3577831

[9] Johnston JW, Briles DE, Myers LE, Hollingshead SK (2006) Mn^2+^-dependent regulation of multiple genes in *Streptococcus pneumoniae* through PsaR and the resultant impact on virulence. Infect Immun 74: 1171–1180. 16428766 10.1128/IAI.74.2.1171-1180.2006 PMC1360317

[10] Karavolos MH, Horsburgh MJ, Ingham E, Foster SJ (2003) Role and regulation of the superoxide dismutases of *Staphylococcus aureus.* . Microbiology 149: 2749–2758. 14523108 10.1099/mic.0.26353-0

[11] Handke LD, Hawkins JC, Miller AA, Jansen KU, Anderson AS (2013) Regulation of *Staphylococcus aureus* MntC expression and its role in response to oxidative stress. PLoS ONE 8: e77874. 24205007 10.1371/journal.pone.0077874 PMC3810276

[12] Kehl-Fie TE, Zhang Y, Moore JL, Farrand AJ, Hood MI, et al (2013) MntABC and MntH contribute to systemic *Staphylococcus aureus* infection by competing with calprotectin for nutrient manganese. Infect Immun 81 (9): 3395–3405. 10.1128/IAI.00420-13 PMC3754211 23817615

[13] Anderson AS, Scully IL, Timofeyeva Y, Murphy E, McNeil LK, et al (2012) *Staphylococcus aureus* manganese transport protein C is a highly conserved cell surface protein that elicits protective immunity against *S. aureus* and *Staphylococcus epidermidis* . J Infect Dis 205: 1688–1696. 22474033 10.1093/infdis/jis272 PMC3348682

[14] Gribenko A, Mosyak L, Ghosh S, Parris K, Svenson K, et al (2013) Three-dimensional structure and biophysical characterization of *Staphylococcus aureus* cell surface antigen–manganese transporter MntC. J Mol Biol 425: 3429–3445. 23827136 10.1016/j.jmb.2013.06.033

[15] Planet PJ, LaRussa SJ, Dana A, Smith H, Xu A, et al (2013) Emergence of the epidemic methicillin-resistant *Staphylococcus aureus* strain USA300 coincides with horizontal transfer of the arginine catabolic mobile element and speG-mediated adaptations for survival on skin. MBio 4: e00889–13. 24345744 10.1128/mBio.00889-13 PMC3870260

[16] Dintilhac A, Alloing G, Granadel C, Claverys JP (1997) Competence and virulence of *Streptococcus pneumoniae*: Adc and PsaA mutants exhibit a requirement for Zn and Mn resulting from inactivation of putative ABC metal permeases. Molecular Microbiology 25: 727–739. 9379902 10.1046/j.1365-2958.1997.5111879.x

[17] Anderton JM, Rajam G, Romero-Steiner S, Summer S, Kowalczyk AP, et al (2007) E-cadherin is a receptor for the common protein pneumococcal surface adhesin A (PsaA) of *Streptococcus pneumoniae* . Microb Pathog 42: 225–236. 17412553 10.1016/j.micpath.2007.02.003

[18] Pimenta FC, Miyaji EN, Arêas AP, Oliveira ML, de Andrade AL, et al (2006) Intranasal immunization with the cholera toxin B subunit-pneumococcal surface antigen A fusion protein induces protection against colonization with *Streptococcus pneumoniae* and has negligible impact on the nasopharyngeal and oral microbiota of mice. Infect Immun 74: 4939–4944. 16861686 10.1128/IAI.00134-06 PMC1539618

[19] Arêas AP, Oliveira ML, Miyaji EN, Leite LC, Aires KA, et al (2004) Expression and characterization of cholera toxin B-pneumococcal surface adhesin A fusion protein in *Escherichia coli*: ability of CTB-PsaA to induce humoral immune response in mice. Biochem Biophys Res Commun 321: 192–196. 15358234 10.1016/j.bbrc.2004.06.118

[20] Diep BA, Phung Q, Date S, Arnott D, Bakalarski C, et al (2014) Identifying potential therapeutic targets of Methicillin-resistant *Staphylococcus aureus* through *in vivo* proteomic analysis. J Infect Dis 209 (10): 1533–1541. 24280367 10.1093/infdis/jit662 PMC3997574

[21] Wolff DG, Castiblanco-Valencia MM, Abe CM, Monaris D, Morais ZM, et al (2013) Interaction of *Leptospira* Elongation Factor Tu with plasminogen and complement factor H: A metabolic Leptospiral protein with moonlighting activities. PLoS ONE 8: e81818. 24312361 10.1371/journal.pone.0081818 PMC3842364

[22] Mulcahy ME, Geoghegan JA, Monk IR, O'Keeffe KM, Walsh EJ, et al (2012) Nasal colonisation by *Staphylococcus aureus* depends upon clumping factor B binding to the squamous epithelial cell envelope protein loricrin. PLoS Pathogens 8 (12): e1003092. 23300445 10.1371/journal.ppat.1003092 PMC3531522

[23] Chagnot C, Listrat A, Astruc T, Desvaux M (2012) Bacterial adhesion to animal tissues: protein determinants for recognition of extracellular matrix components. Cellular Microbiology 14 (11): 1687–1696. 22882798 10.1111/cmi.12002

[24] Geoghegan JA, Ganesh VK, Smeds E, Liang X, Höök M, et al (2010) Molecular characterization of the interaction of Staphylococcal Microbial Surface Components Recognizing Adhesive Matrix Molecules (MSCRAMM) ClfA and Fbl with Fibrinogen. The Journal of Biological Chemistry 285 (9): 6208–6216. 20007717 10.1074/jbc.M109.062208 PMC2825416

[25] Mölkänen T, Tyynelä J, Helin J, Kalkkinen N, Kuusela P (2002) Enhanced activation of bound plasminogen on *Staphylococcus aureus* by Staphylokinase. FEBS Lett 517 (1–3): 72–78. 12062412 10.1016/s0014-5793(02)02580-2

[26] Carneiro CRW, Postol E, Nomizo R, Reis LFL, Brentani RR (2004) Identification of enolase as a laminin-binding protein on the surface of *Staphylococcus aureus.* . Microbes and Infection 6: 604–608. 15158195 10.1016/j.micinf.2004.02.003

[27] Barbosa AS, Monaris D, Silva LB, Morais ZM, Vasconcellos SA, et al (2010) Functional characterization of LcpA, a surface-exposed protein of *Leptospira* spp. that binds the human complement regulator C4BP. Infect Immun 78: 3207–3216. 20404075 10.1128/IAI.00279-10 PMC2897400

[28] Castiblanco-Valencia MM, Fraga TR, Silva LB, Monaris D, Abreu PA, et al (2012) Leptospiral immunoglobulin-like proteins interact with human complement regulators factor H, FHL-1, FHR-1, and C4BP. J Infect Dis 205: 995–1004. 22291192 10.1093/infdis/jir875

[29] Cameron CE (2003) Identification of a *Treponema pallidum* laminin-binding protein. Infect Immun 71: 2525–2533. 12704124 10.1128/IAI.71.5.2525-2533.2003 PMC153296

[30] Choy HA, Kelley MM, Chen TL, Møller AK, Matsunaga J, et al (2007) Physiological osmotic induction of *Leptospira interrogans* adhesion: LigA and LigB bind extracellular matrix proteins and fibrinogen. Infect Immun 75: 2441–2450. 17296754 10.1128/IAI.01635-06 PMC1865782

[31] Lin YP, Chang YF (2007) A domain of the *Leptospira* LigB contributes to high affinity binding of fibronectin. Biochem Biophys Res Commun 362: 443–448. 17707344 10.1016/j.bbrc.2007.07.196

[32] Lin YP, Chang YF (2008) The C-terminal variable domain of LigB from *Leptospira* mediates binding to fibronectin. J Vet Sci 9: 133–144. 18487934 10.4142/jvs.2008.9.2.133 PMC2839090

[33] Lin YP, Raman R, Sharma Y, Chang YF (2008) Calcium binds to leptospiral immunoglobulin-like protein, LigB, and modulates fibronectin binding. J Biol Chem 283: 25140–25149. 18625711 10.1074/jbc.M801350200

[34] Lin YP, Lee DW, McDonough SP, Nicholson LK, Sharma Y, et al (2009) Repeated domains of *Leptospira* immunoglobulin-like proteins interact with elastin and tropoelastin. J Biol Chem 284: 19380–19391. 19473986 10.1074/jbc.M109.004531 PMC2740563

[35] Lin YP, Greenwood A, Nicholson LK, Sharma Y, McDonough SP, et al (2009) Fibronectin binds to and induces conformational change in a disordered region of leptospiral immunoglobulin-like protein B. J Biol Chem 284: 23547–23557. 19581300 10.1074/jbc.M109.031369 PMC2749129

[36] Lin YP, Greenwood A, Yan W, Nicholson LK, Sharma Y, et al (2009) A novel fibronectin type III module binding motif identified on C-terminus of *Leptospira* immunoglobulin-like protein, LigB. Biochem Biophys Res Commun 389: 57–62. 19699715 10.1016/j.bbrc.2009.08.089 PMC2804977

[37] Lin YP, McDonough SP, Sharma Y, Chang YF (2010) The terminal immunoglobulin-like repeats of LigA and LigB of *Leptospira* enhance their binding to gelatin binding domain of fibronectin and host cells. PLoS ONE 5: e11301. 20585579 10.1371/journal.pone.0011301 PMC2892007

[38] Verma A, Brissette CA, Bowman AA, Shah ST, Zipfel PF, et al (2010) Leptospiral endostatin-like protein A is a bacterial cell surface receptor for human plasminogen. Infect Immun 78: 2053–2059. 20160016 10.1128/IAI.01282-09 PMC2863546

[39] Scully IL, Liberator PA, Jansen KU, Anderson AS (2014) Covering all the bases: preclinical development of an effective *Staphylococcus aureus* vaccine. Frontiers in Immunology 5 (art 109): 1–7. 10.3389/fimmu.2014.00109 PMC3970019 24715889

[40] Shiri T, Nunes MC, Adrian PV, Van Niekerk N, Klugman KP, et al (2013) Interrelationship of *Streptococcus pneumoniae*, *Haemophilus influenzae* and *Staphylococcus aureus* colonization within and between pneumococcal-vaccine naïve mother-child dyads. BMC Infectious Diseases 13: 483–492. 24134472 10.1186/1471-2334-13-483 PMC4015913

[41] Mina MJ, McCullers JA, Klugman KP (2014) Live attenuated influenza vaccine enhances colonization of *Streptococcus pneumoniae* and *Staphylococcus aureus* in mice. mBio 5(1): e01040–13. 24549845 10.1128/mBio.01040-13 PMC3944816

[42] Kuusela P, Saksela O (1990) Binding and activation of plasminogen at the surface of *Staphylococcus aureus* . European journal of biochemistry 193: 759–765. 1701146 10.1111/j.1432-1033.1990.tb19397.x

[43] Kuusela P, Ullberg M, Saksela O, Kronvall G (1992) Tissue-type plasminogen activator-mediated activation of plasminogen on the surface of group A, C, and G streptococci. Infect Immun 60: 196–201. 1370273 10.1128/iai.60.1.196-201.1992 PMC257522

[44] Wistedt AC, Ringdahl U, Müller-Esterl W, Sjöbring U (1995) Identification of a plasminogen-binding motif in PAM, a bacterial surface protein. Molecular microbiology 18: 569–578. 8748039 10.1111/j.1365-2958.1995.mmi_18030569.x

[45] Brissette CA, Haupt K, Barthel D, Cooley AE, Bowman A, et al (2009) *Borrelia burgdorferi* infection-associated surface proteins ErpP, ErpA, and ErpC bind human plasminogen. Infect Immun 77: 300–306. 19001079 10.1128/IAI.01133-08 PMC2612283

[46] Zautner AE, Krause M, Stropahl G, Holtfreter S, Frickmann H, et al (2010) Intracellular persisting *Staphylococcus aureus* is the major pathogen in recurrent tonsillitis. PLoS ONE 5(3): e9452. 20209109 10.1371/journal.pone.0009452 PMC2830486

[47] Law RH, Abu-Ssaydeh D, Whisstock JC (2013) New insights into the structure and function of the plasminogen/plasmin system. Current opinion in structural biology 23: 836–841. 24252474 10.1016/j.sbi.2013.10.006

[48] Halkier T (1991) Mechanisms in blood coagulation, fibrinolysis and the complement system. Cambridge University Press.

[49] Grosskinsky S, Schott M, Brenner C, Cutler SJ, Kraiczy P, et al (2009) *Borrelia recurrentis* employs a novel multifunctional surface protein with anti-complement, anti-opsonic and invasive potential to escape innate immunity. PLoS ONE 4: e4858. 19308255 10.1371/journal.pone.0004858 PMC2654920

[50] Lähteenmäki K, Kuusela P, Korhonen TK (2001) Bacterial plasminogen activators and receptors. FEMS Microbiol Rev. 25 (5): 531–552. 10.1111/j.1574-6976.2001.tb00590.x 11742690

[51] Koch TK, Reuter M, Barthel D, Böhm S, van den Elsen J, et al (2012) *Staphylococcus aureus* proteins Sbi and Efb recruit human plasmin to degrade complement C3 and C3b. PLoS ONE 7 (10): e47638. 23071827 10.1371/journal.pone.0047638 PMC3469469

[52] Furuya H, Ikeda R (2009) Interaction of triosephosphate isomerase from the cell surface of *Staphylococcus aureus* and α-(1->3)-mannooligosaccharides derived from glucuronoxylomannan of *Cryptococcus neoformans* . Microbiology 155 (Pt 8): 2707–2713. 10.1099/mic.0.028068-0 PMC2885673 19423633

[53] Feng YE, Chen C-J, Su L-H, Hu S, Yu JE, et al (2008) Evolution and pathogenesis of *Staphylococcus aureus*: lessons learned from genotyping and comparative genomics. FEMS Microbiology Reviews 32: 23–37. 17983441 10.1111/j.1574-6976.2007.00086.x

