# Correction: *Staphylococcus aureus* Manganese Transport Protein C (MntC) Is an Extracellular Matrix- and Plasminogen-Binding Protein

**DOI:** 10.1371/journal.pone.0121192

**Published:** 2015-03-25

**Authors:** The PLOS ONE Staff

There is an error in the published article. Paragraph eight of the Introduction incorrectly states that ClfA was the first fibronectin-binding protein isolated in *S. aureus*. The Introduction should state that ClfA was the first fibrinogen γ-chain-binding protein isolated in *S. aureus*.
